# Association between *Helicobacter pylori* Infection and Occurrence of Anemia among Pregnant Women Attending Antenatal Care in Kulito Health Center, Halaba Zone, South Ethiopia, 2018

**DOI:** 10.1155/2020/6574358

**Published:** 2020-07-24

**Authors:** Bahredin Abdella, Mohammed Ibrahim, Iyasu Tadesse, Kalkidan Hassen, Mekonnin Tesfa

**Affiliations:** ^1^Department of Nursing, College of Health Science, Werabe University, Werabe, Ethiopia; ^2^Department of Biomedical Sciences, Institute of Health, Jimma University, Jimma, Ethiopia; ^3^Department of Biomedical Sciences, Faculty of Health Sciences, Woldia University, Woldia, Ethiopia

## Abstract

**Background:**

Anemia in pregnancy is defined as a hemoglobin (Hb) concentration of less than 11 grams (gm)/deciliter (dl) in venous blood. Globally, it affects 1.62 billion people. In developing countries, anemia is a major cause of maternal and child morbidity and mortality. Globally, anemia contributes to 20% of all maternal deaths. Nearly 50% of the world's population is estimated to be infected with *Helicobacter pylori* (HP). High prevalence of HP among pregnant women was also reported in developing countries than developed ones. The association between HP infection and occurrence of anemia is not well known in Ethiopia. Therefore, the aim of this study was to determine the association between anemia and *Helicobacter pylori* infection among pregnant women attending antenatal care follow-up in Kulito Health Center, Halaba Zone, South Ethiopia.

**Methods:**

Institution-based cross-sectional study was employed. Systematic random sampling procedure was employed to select 236 pregnant women who attended antenatal care at Kulito Health Center. An interviewer-administered questionnaire supplemented by laboratory tests was used to obtain the data. The collected data were analyzed by using SPSS version 20.0.

**Results:**

The prevalence of anemia among antenatal care attendant pregnant women of Kulito Health Center was 27.5% with 36 (15.2%) of mild, 29 (12.3%) of moderate, and no severe cases of anemia. The overall prevalence of HP infection among study participants was found to be 129 (54.7%) (95% CI: 47.9–61.4). Factors significantly associated with anemia were presence of HP infection (AOR = 3.064, 95% CI: 1.336 7.027), low interpregnancy gap (AOR = 2.863, 95% CI: 1.245–6.582), being on the third trimester (AOR = 6.457; 95% CI: 1.276–32.729), and mid-upper arm circumference (MUAC) level <21 cm (AOR = 2.595, 95% CI: 1.044–6.450).

**Conclusion:**

This study revealed that anemia and HP infection were highly prevalent among pregnant women attending the antenatal follow-up clinic in Kulito Health Center. HP infection, low interpregnancy gap, being on the third trimester, and MUAC less than 21 cm were the independent factors associated with anemia. *Recommendation*. Pregnant women should be aware that anemia is a problem that can be prevented by early prevention and treatment of HP infection and undernutrition, using family planning to widen the interpregnancy gap. Further experimental studies are warranted to determine the cause and effect of the association between anemia and HP infection.

## 1. Introduction

Anemia is a condition in which the number of RBCs or their oxygen capacity is insufficient to meet physiological needs [1]. Anemia in pregnancy is defined as a decreased Hb concentration less than 11 gm/dl in venous blood. During pregnancy, anemia is considered as severe when Hb concentration is less than 7.0 gm/dl, moderate when Hb falls between 7.0 and 9.9 gm/dl, and mild when Hb concentration is between 10.0 and 11 gm/dl [2].

Anemia is one of the most common nutritional deficiency diseases observed globally and affects more than a quarter of the world's population of which children below the age of five and pregnant women are highly vulnerable groups [3]. Half of all the cases of anemia can be attributed to iron deficiency (ID) [4]. Nearly half of all pregnant women have been affected by anemia globally. The burden of anemia in pregnant mothers living in developing countries is higher (55%) than the developed ones (19%) [5]. It is also a significant public health problem in Ethiopia [6].

Despite iron supplementation for all pregnant mothers in our country, many cross-sectional studies conducted in different parts of Ethiopia showed the significant magnitude of anemia among pregnant women: 25.2%, in Aymba, northwest Ethiopia [7], 56.8% in Gode, western Ethiopia [8], 61.6% in Boditi, southern Ethiopia [9], 32.8% in Arba Minch, southern Ethiopia [2], 27.2% in Butajira, South Ethiopia [10], and 27.9% in Harerge, southeast Ethiopia [6].

Globally, anemia contributes to 20% of maternal death [11]. Anemia in pregnancy is not only a risk factor of maternal mortality but is also harmful for the fetus due to increased risk of adverse birth outcomes such as intrauterine growth retardation, premature birth, and low birthweight (LBW) [3]. In developing countries, the risk factors of anemia during pregnancy are multifactorial. This includes nutritional deficiencies, parasitic infection, and sociodemographic and economic status of the mothers [4]. Another factor that is believed to contribute to the occurrence of anemia in pregnancy is a defect in gastric absorption and utilization of dietary or supplemental iron due to HP infection [12]. HP infection is the most common infection worldwide and is associated with simple dyspepsia, heartburn, and peptic ulcer diseases, most commonly leading to upper gastrointestinal bleeding and, ultimately, to the severe complication of gastric malignancy [13].

Nearly 50% of the world's population is estimated to be infected with HP [14]. High prevalence of HP among pregnant women was also reported in developing countries than developed ones [15]. Many studies conducted in different parts of the world showed the significant magnitude of HP infection: 33.3%, in Addis Ababa [16], 50.7% in Jinka [17], 52.4% in Butajira [18], 71% in Jijiga [19], 83.5% in Hawassa [20], and 54.2% in Iran [12]. High prevalence of HP infection has been observed among pregnant women, which was associated with increased risk of anemia [21]. It is hypothesized that HP associated with anemia is caused by both compromised absorption of bioavailable iron in the context of hypochlorhydria and the competing iron demands of HP and the host [22]. Bleeding through peptic ulcers and other gastric lesions due to bacterial irritation of the gastric mucosa also contribute to the occurrence of anemia [12].

A systematic review and meta-analysis found higher prevalence of IDA in HP-infected subjects than uninfected ones [21]. The study conducted in Turkey in the general population showed that serum hemoglobin level was significantly reduced among individuals infected with HP relative to uninfected patients [23]. The cross-sectional prospective study conducted in Butajira showed the prevalence of anemia among HP-infected patients was 30.9% and while 22.5% among uninfected patients [18].

The experimental study done in India found higher prevalence of IDA in HP-infected pregnant women than uninfected ones. Additionally, the study concluded that high prevalence of HP infection was seen in pregnant women suffering from IDA and eradication of the infection by triple drug therapy during the third trimester enhanced the response to oral iron and folic acid supplementation [24]. Another cross-sectional study conducted in Iran among 180 pregnant women showed a significant and adverse correlation between HP infection and hemoglobin level [12]. Even though many studies found the association of anemia and HP infection, a study done in Sudan failed to find the association between HP infection and anemia [25]. The study conducted among children in Butajira also showed the absence of significant difference in the prevalence of anemia among HP-infected and noninfected children [26]. The outcome of this study may help the stakeholders to take measures to reduce this public health issue and policy makers to consider HP infection as the risk factor for anemia.

## 2. Materials and Methods

### 2.1. Study Design, Area, and Period

Institution-based cross-sectional study was conducted in Kulito Health Center, Kulito town, from April to May 9, 2018. Kulito town is the capital of Halaba Zone which is found in SNNPR, Ethiopia. It is located about 245 km far from the capital city of Addis Ababa and 90 km away in the northwest direction from the capital of SNNPR, Hawassa.

### 2.2. Population and Eligibility Criteria

The study population was all pregnant women who have antenatal care follow-up at Kulito Health Center during the study period. The inclusion criteria were all pregnant women who were attending the antenatal care follow-up clinic in Kulito Health Center during the data collection period. Pregnant women who have active bleeding or history of bleeding during the current pregnancy, who have taken treatment for HP infection in the last two months, who are known to have chronic diseases (heart failure, renal failure, liver disease, diabetic, HIV AIDS, cancer, and bleeding disorder), and who are seriously ill were excluded from the study.

### 2.3. Sample Size Determination

The sample size was determined based on the single population proportion formula using *Z*2 × *p* × *q*/*d*2 with a 95% confidence interval and 5% margin of error [27]. Sample size was calculated by using 27.6% anemia prevalence from nearby the study area [10]. Thus, the total sample size was 307, and by adding 10% of nonrespondent rate, it was 338. Since the total ANC followers in the study area were 860 which were less than 10,000, we used the correction formula to calculate the final sample size. Therefore, the final sample size for this study was 241. The systematic random sampling (lottery) method was used to select the first study participant, and a systematic random sampling method (*k* = *N*/*n* = 860/241 = 3.56 = 4) was used to select the rest. Thus, every second (*k* = 4), pregnant women who meet our inclusion criteria were selected.

### 2.4. Operational Definitions

Anemia in pregnancy: it is when the hemoglobin value for a pregnant woman is less than 11 gm/dl, irrespective of her gestational age [27]. Mild anemia: venous blood Hb concentration is 10–10.9 gm/dl [27]. Moderate anemia: venous blood Hb concentration is 7–9.9 gm/dl [27]. Severe anemia: venous blood Hb concentration is <7 gm/dl [27]. Public health importance of anemia: it is a mild public health problem when prevalence of anemia is <20%; a moderate public health problem when the prevalence of anemia is between 20 and 40%; and a severe public health problem when the prevalence of anemia is >40% [6]. Primary and above: the context of the primary and above educational level expresses the study participants who have attended formal education. Other health-related variables: variables like sociodemographic characteristics, nutritional status, IP infection, and obstetric history of the study participants. Interpregnancy gap: it is defined as the number of years between the previous live birth and conception of the current pregnancy. Low interpregnancy gap: the interpregnancy gap is less than 2 years.

Mid-upper arm circumference (MUAC) is a measurement of the circumference of the upper arm at the midpoint between the olecranon and acromion processes. It is normal when MUAC level is ≥21 cm; undernutrition when MUAC level is <21 cm [28].

### 2.5. Data Collection Procedures

Data were collected by two midwives and two laboratory technicians and overseen by the supervisor. Data collectors have measured the MUAC level to the nearest 0.1 cm. A structured pretested interviewer-administered questionnaire was used to obtain sociodemographic information and present and past obstetric history in pregnant women [10, 15]. Data were collected through face-to face interview. The questionnaire was initially developed in English and translated into the working language (Amharic) and then retranslated back to English to maintain its consistency.

### 2.6. Specimen Collection and Processing

The specimen collection was carried out by two trained laboratory technicians. Each step of specimen collection, processing, and analysis was supervised by experienced and trained laboratory technologist supervisors. Blood for hematocrit/packed cell volume (PCV) measurement was done based on the Standard Operational Procedures (SOPs). Venous blood samples (4 ml from each participant) were collected by laboratory technicians in Vacutainer tubes (BD, USA) containing an anticoagulant (EDTA (ethylenediaminetetraacetic acid)). Hb was measured using BC-3000 plus Auto Hematology Analyzer (Mindray; Nansha, Shenzhen 518057, China). The golden standard method for detecting HP infection status is *H. pylori* stool antigen testing kit. However, due to lack of budget, we had used the *H. pylori* antibody test strip for testing of maternal HP infection status. *H. pylori* antibody test strip is a qualitative membrane-based immunoassay for the detection of HP antibodies in serum or plasma. In this test procedure, anti-human IgG was immobilized in the test line region of the test. One drop of whole blood and three drops of buffer were added on the appropriate space of the kit based on the SOPs. After the specimen and buffer were added, the specimen reacts with HP antigen-coated particles in the test. This mixture migrates chromatographically along the length of the test and interacts with immobilized anti-human IgG. If the specimen contains HP antibodies, a colored line will appear in the test line region indicated a positive result. If the specimen does not contain HP antibodies, a colored line will not appear in this region indicating a negative result. To serve as a procedural control, a colored line always appeared in the control line region, indicating that proper volume of the specimen has been added, and membrane wicking has occurred [20].

Stool sample processing for direct stool examination (wet smear): a drop of normal saline was put on the cleaned microscope slides, and a small amount of the stool specimen was taken with a wooden stick and mixed with saline and was examined as soon as possible (within 30 minutes of passage), and on soft/formed stool within 60 minutes of passage, helminth ova was examined using the 10x objective, and cysts and trophozoites were examined using the 40x objective; this aids to detect certain protozoa trophozoites with their identification [16].

### 2.7. Data Quality Control

Before the actual data collection, the questionnaire was pretested on 12 pregnant women in Kulito primary hospital nearby the health center in order to estimate the time needed to collect data, and the questionnaires were modified accordingly. Training was given for data collectors before data collection regarding the purpose of the study, interview, and ethical issues during data collection. Data collectors were instructed to completely fill the questionnaire. The data were checked for completeness and consistency throughout the data collection period.

### 2.8. Data Analysis Procedures

Data were checked for completeness, coded, and entered into EPI-data version 3.1 (Odense, Denmark) and then transferred to SPSS version 20.0 (IBM, Armonk, NY, USA) for analysis. Frequencies, means, and percentage were used to give a clear picture of sociodemographic variables. Bivariate analysis was performed to select candidate variables at *P* value ≤ 0.25. The variables that have statistically significant associations with the outcome variable in the bivariate analysis were further considered as a candidate for the backward stepwise multiple logistic regression model to control the effect of confounding variables. Multivariate analysis was carried out to declare variables that are independently associated with anemia, and adjusted odds ratios (AORs) were used to indicate the strength of association between dependent and independent variables. Finally, those variables with *P* value < 0.05 in the final model were considered as statistically significant.

### 2.9. Ethical Consideration

Ethical clearance was obtained from the Institutional Review Board of Jimma University, with ethical approval reference number IHRPGD/242/2018, and letter of cooperation was obtained from the Halaba Zone Health Department and Kulito HC Administration prior to the kickoff of the study. The objectives, benefits, and risks of the study were explained to the participants, and verbal informed consent was obtained from each participant. Participant's confidentiality was maintained, and anonymity was insured by using codes instead of names and any personal identifier of the participants. However, those with anemia, HP infections, and IP infection were referred to concerned health personnel for appropriate intervention.

## 3. Results

### 3.1. Sociodemographic Characteristics

Out of the total sample size (241), 236 pregnant women were included with a respondent rate of 97.93%. The mean age of the women was 26.9 ± 6.3 (SD) with a range of 16–42 years. Nearly half 109 (46.2%) of the women were within the age group 25–32 years. A majority of the study participants were married 222 (94.1%), and few of them 14 (5.9 %) were divorced. More than half of the participants (59.3%) were urban residents (Table 1).

### 3.2. Obstetric History, Nutritional Characteristics, and Intestinal Parasite Status of the Study Participants

Among all pregnant women, 152 (64.4%) were multigravida, and 84 (35.6%) were primigravida. A majority of the women 104 (68.4%) had interpregnancy gap of more than two years. More than half of the pregnant women 123 (52.1%) were in their third trimester followed by those in their second 74 (31.4%) and in their first 39 (16.5%). Most (93.6%) of the participants had no history of hyperemesis gravidarum during the current pregnancy. Some of the study participants 68 (28.8%) were taking iron folate during this pregnancy, but a majority 168 (71.2%) of them did not take iron folate. More than half 147 (62.3%) of the participants had meal ≤ three times a day, and 89 (37.7%) had four times and above. Nutritional status was evaluated by MUAC, and 236 of the respondents were measured; 44 (18.6%) had MUAC of less than 21 cm, and 192 (81.4%) had MUAC within normal limits (≥21 cm) (Table 2).

### 3.3. Prevalence of HP Infection

**Figure 1:**
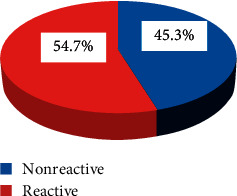
Prevalence of HP infections among pregnant women in Kulito Health Center, SNNPR, Ethiopia, 2018 (*n* = 236).

Based on the serology IgG test for diagnosis of HP, 129 participants (54.7%) were found positive. The prevalence of HP infection is presented in Figure 1.

### 3.4. Prevalence of Anemia

**Figure 2:**
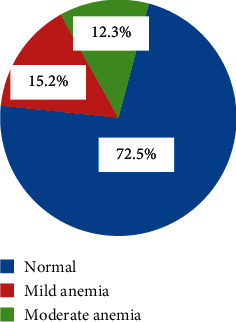
Prevalence of anemia among pregnant women in Kulito Health Center, SNNPR, Ethiopia, 2018 (*n* = 236).

The participants' Hb level was used to determine the presence or absence and stage of anemia. Hb concentration of the study participants ranged from 7 to 14 gm/dl, with a mean (±SD) of 11.45 ± 1.58 gm/dl. The overall burden of anemia among the study participants was 65 (27.5%) with 15.2% mild, 12.3% moderate, and no severe cases (Figure 2).

### 3.5. Anemia and *Helicobacter pylori*

**Figure 3:**
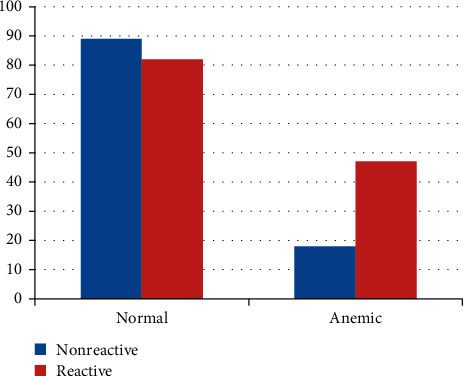
Anemia and HP infection among the study participants in Kulito Health Center, SNNPR, Ethiopia, 2018 (*n* = 236).

The overall prevalence of HP infection among study participants was 129 (54.7%). The magnitude of anemia was high among HP-infected women than noninfected ones (Figure 3).

### 3.6. Anemia and Other Related Factors

The prevalence of anemia was high among pregnant women who were within the age of 32–42 years (18/53 (43%)), unemployed (46/137 (33.6%)), rural residents (37/96 (38.5%)), and illiterate (27/78 (34.6%)). The magnitude of anemia increased among study participants with the family size of more than or equal to five with low monthly family income. The prevalence of anemia was also found to increase as the gestational age increased, showing the highest prevalence in the third trimester (31.5%) compared with the second (30%) and first (10.3%) trimester. Women with IPG less than two years showed more prevalence of anemia (40.8%) than those with an IPG greater than or equal to two years (22.1%). The prevalence of anemia was high among pregnant women whose MUAC was less than 21 cm (52.3%). However, the magnitude of anemia was high in women who had no IP infection (45/65 (69.2%)) than infected ones (20/65 (30.8%)) (Table 3).

### 3.7. The Relationship between Anemia and Other Related Factors

The variables that showed statistically significant association (at *P* value ≤ 0.25) in the bivariate analysis were transferred and further analyzed in multivariable logistic regression to adjust for potential confounders and to identify predictors of anemia. In multivariable logistic regression, variables with *P* value less than 0.05 were considered as independent factors for anemia. The model was tested for multicollinearity (VIF = 1.088–2.022), and Hosmer–Lemeshow test was used for goodness of fit (*P*=0.810); as a result, the model was fit, and no multicollinearity existed. In multivariate logistic regression analysis, IPG, gestational age, and MUAC level were variables independently associated with anemia (Table 4).

Pregnant women with IPG less than two years were 2.8 times (AOR = 2.863, 95% CI: 1.245–6.582) more likely to suffer from anemia when compared to having IPG greater than two years. Another important predictor was gestational age being on the third trimester which was 6.5 times (AOR = 6.457; 95% CI: 1.276–32.729) more likely to suffer from anemia when compared with those on the first trimester. Most importantly, MUAC level less than 21 cm was strongly associated with anemia. Pregnant women with MUAC level less than 21 cm were 2.6 times (AOR = 2.595, 95% CI: 1.044–6.450) more likely to develop anemia as compared with participants with MUAC level greater than 21 cm.

### 3.8. The Relationship between Anemia and HP Infection

Pregnant women with HP infection were 3 times (AOR = 3.064, 95% CI: 1.336–7.027) higher to develop anemia as compared with those without HP infection (Table 5).

## 4. Discussion

The current study assessed the association between HP infection and occurrence of anemia among pregnant women attending antenatal care at Kulito Health Center, Halaba Zone, South Ethiopia. This study found significant association between anemia and HP infection (AOR = 3.064, 95% CI = 1.336–7.027). Pregnant women with HP infection were more likely to have anemia than those without HP infection. This finding is in agreement with the previous study done among pregnant women in Addis Ababa [16], Iran [12], India [24], and Turkey [23]. Similarly, the study conducted in Butajira among dyspeptic nonpregnant patients also found significant association between anemia and HP infection [20]. However, the study conducted in Butajira among children and Sudan among pregnant women was failed to found association between anemia and HP infection [25, 26].

The probable finding of these results could be due to some possible mechanisms by which HP affects iron metabolism by decreased absorption resulting from chronic gastritis, decreased gastric juice ascorbic acid concentration, increased hepcidin production associated with HP gastritis, uptake of iron by HP for growth, and decreased availability of iron by sequestration of iron in lactoferrin in the gastric mucosa and bacterium host competition for dietary iron supply [22]. Another explanation most commonly offered for this relationship could be also based upon the development of HP-associated chronic gastritis with resultant achlorhydria and reduced ascorbic acid secretion leading to reduced intestinal iron absorption [18]. Besides, an association between anemia and HP includes occult blood loss from erosive gastritis and sequestration and utilization of iron by the organism [12].

The burden of prenatal anemia is widely recognized as a major public health problem throughout the world, particularly in developing countries [9]. Because of blood volume expansion and increased iron demand of the fetus and the mother, hemoglobin level altered dramatically during the course of pregnancy [24]. This study noted that the prevalence of anemia in this study population was found to be 27.5% (95% CI = 22.0, 33.5). It is of moderate public health significance according to the WHO criterion, which means the magnitude of anemia is within the range of 20–39.9% [29, 30].

The overall prevalence of anemia of this study is comparable with the former studies from various parts in Ethiopia, such as 27.6% from Butajira General Hospital, southern Ethiopia, 27.9% from Harerge, southeast Ethiopia, and 29.1% from Uganda [6, 10, 31]. The result was slightly higher than the previous local reports from Aymba HC, northwest Ethiopia, 25.2% [7]. However, this report was lower than another study such as 61.6% was reported from Boditi, southern Ethiopia, 56.8% from Gode, southeast Ethiopia, 32.8% from Arba Minch, southern Ethiopia, and 31% from Iran [2, 8, 9, 12]. Such magnitude differences may be due to differences in inclusion and exclusion criterion and dietary characteristics of the study participants between the studies. The above study included pregnant women with known chronic illness and bleeding, but in our study, they were excluded.

Systematic review and meta-analysis study found that worldwide prevalence of HP infection in the general population ranges from 25% to 94% [21]. Another systematic review and meta-analysis conducted in 2017 indicates the global prevalence of HP infection in pregnant women was 46% (23).The prevalence of HP infection in this study population was found to be 54.7% (95% CI: 47.9–61.4).

This prevalence of HP infection is consistent with studies conducted in Jinka and Butajira among the general population which reported prevalence of HP infection to be 50.7% and 52.4%, respectively [17, 18]. The result of this study is also similar with the study done in Iran which found prevalence of HP infection to be 54.2% among pregnant women [12].

The study conducted among the general population in Jijiga and Hawassa reported high prevalence of HP infection as compared with this study which found prevalence of HP infection to be 71% and 83.3%, respectively [19, 32]. However, the result of this study is higher than the study conducted among pregnant women in Addis Ababa [16]. The difference of the magnitude of HP infection might be due to the difference in sample size, sociodemography, and especially laboratory methods they used.

Anemia is a problem that is caused by several factors. The present study found IPG as a predictor of anemia, and thus, pregnant women with <2 years of IPG were 3 times more likely to suffer anemia when compared with those having ≥2 years of IPG. Similar findings were also reported from the studies conducted at Arba Minch, Asosa Zone, and Butajira [2, 10, 11]. In contrast, the study conducted in Boditi showed that anemia was more prevalent in women with IPG greater than or equal to two years (87.5%) than those with less than two years (12.5%) [9]. Appropriate time after each pregnancy for recovery and replenishment of nutrient stores requires 2–5 years. Pregnancy with a short birth interval leads to IDA as iron requirements are substantially higher than the average [1]. The risk of maternal nutritional depletion increases with closed birth intervals [11].

Gestational age was also another factor significantly associated with anemia in this study. Pregnant women in the third trimester pregnancy were 6.5 times more likely to develop anemia as compared with those in the first trimester pregnancy. Similarly the study done in Boditi, Harerge, and Gode showed significant association of anemia with the third trimester of gestational age [6, 8, 9]. In contrast, the study conducted in Aymba HC, Amhara region, and Asosa Zone failed to find association of anemia with third trimester pregnancy [11, 20].

This could be due to the fact that when the gestational age increases, the mother becomes weak, and iron in the blood is shared with the fetus in the womb. Another factor is increasing of total blood volume as the gestational age increases.

Another variable significantly associated with anemia was MUAC level. MUAC less than 21 cm was found to increase the risk of developing anemia. The current study showed that pregnant women with MUAC<21 cm had 2.6 times more risk of developing anemia than those with MUAC≥21 cm. This finding is consistent with a study done in Asosa Zone and Gode [8, 11]. However, the study conducted in Mekelle failed to find the significant association of MUAC<21 cm and anemia [28]. Pregnancy is the most nutritionally demanding time in a woman's life, which increases the vulnerability of mothers for poor micronutrient reserve, including iron. Undernutrition impaired production of iron transport proteins and increased depletion of stored iron may contribute for the occurence of anemia among undernourished pregnant women [7].

## 5. Conclusion

According to the present study, the overall prevalence of anemia among women attending the antenatal care clinic in Kulito Health Center was 27.5% (95% CI = 22.0, 33.5). Anemia is a moderate public health problem in Kulito Health Center. The overall prevalence of HP infection in this study population was found to be 54.7% (95% CI: 47.9–61.4). HP infection, MUAC level, IPG, and gestational age of being in the third trimester were significantly associated with anemia. Pregnant women should be aware that anemia is a problem that can be prevented by early prevention and treatment of HP infection and undernutrition, using family planning to widen the interpregnancy gap. Therefore, a means to increase their awareness about family planning and prevention and treatment of HP infection and undernutrition should be considered. Further experimental studies are warranted to determine the cause and effect of the association between anemia and HP infection.

## Figures and Tables

**Table 1:** Sociodemographic characteristics of the study participants in Kulito Health Center, SNNPR, Ethiopia, 2018 (*n* = 236).

Characteristics		Frequency, *N*	Percentage (%)

Age	16–24 years	80	33.9
	25–32 years	109	46.2
	33–42 years	47	19.9
Marital status	Married	222	94.1
	Divorced	14	5.9
Educational status	Primary and above	158	66.9
	Illiterate	78	33.1
Occupational status	Employed	99	42
	Unemployed	137	58
Family income	≥2000 birr	88	37.3
	<2000 birr	148	62.7
Family size	≤4 people	142	60.2
	≥5 people	94	39.8
Residency	Urban	140	59.3
	Rural	96	40.7
Hand wash before food	Yes	226	95.8
	No	10	4.2
Hand wash after toilet	Yes	201	No
	No	35	14.8
Water source	Pipe	214	90.7
	River and others	22	9.3

**Table 2:** Obstetric history and nutritional characteristics of the participants in Kulito Health Center, SNNPR, Ethiopia, 2018 (*n* = 236).

Variables	Categories	Frequency, *N*	Percentage (%)

Gravidity	Primigravida	84	35.6
	Multigravida	152	64.4
Gestational age	1^st^ trimester	40	16.5
	2^nd^ trimester	74	31.4
	3^rd^ trimester	132	52.1
Interpregnancy gap for multigravida	<2 years	48	31.6
	≥2 years	104	68.4
Iron pills taken during this pregnancy	Yes	68	28.8
	No	168	71.2
Frequency of diet per day	≥4 times	89	37.7
	≤3 times	147	62.3
Mid-upper arm circumference (MUAC)	≥21 cm	192	81.4
	<21 cm	44	18.6
Intestinal parasite	Yes	49	20.2
	No	187	79.8

**Table 3:** Anemia and other related factors among study participants in Kulito Health Center, SNNPR, Ethiopia, 2018 (*n* = 236).

Variables		Hemoglobin status		Total *n* (%)
		Anemic *n* (%)	Normal *n* (%)	

Age (years)	16–24	25 (31.3)	55 (68.8)	80 (33.9)
	24–32	22 (20.2)	87 (79.8)	109 (46.2)
	32–42	18 (38.3)	29 (61.7)	47 (19.9)
Occupation	Employed	19 (19.2)	80 (80.8)	99 (42)
	Unemployed	46 (33.6)	91 (66.4)	137 (58)
Educational status	Primary and above	38 (24)	120 (76)	158 (67)
	Illiterate	27 (34.6)	51 (65.4)	78 (33)
Family income	≥2000 birr	18 (20.7)	69 (78.3)	87 (36.85)
	<2000 birr	47 (31.5)	102 (68.5)	149 (63.15)
Family size (people)	4 and less	34 (23.9)	108 (76.1)	142 (60.2)
	5 and above	31 (33)	63 (67)	94 (39.8)
Residency	Urban	28 (20)	112 (80)	140 (59.3)
	Rural	37 (38.5)	59 (61.5)	96 (40.7)
Hand wash after toilet	Yes	50 (24.9)	151 (75.1)	201 (85.2)
	No	15 (42.9)	20 (57.1)	35 (14.8)
Gravidity	3 and less	38 (23.45)	124 (75.55)	162 (68.65)
	≥4	27 (36.5)	47 (63.5)	74 (31.35)
Gestational age	1^st^ trimester	4 (10.3)	35 (89.7)	39 (16.5)
	2^nd^ trimester	21 (30)	49 (70)	70 (29.7)
	3^rd^ trimester	40 (31.5)	87 (68.5)	127 (53.8)
IPG (years)	≥2	23 (22.1)	81 (77.9)	104 (68)
	<2	20 (40.8)	29 (59.2)	49 (32)
Taking iron foliate	Yes	15 (22.1)	53 (77.9)	68 (28.8)
	No	50 (29.8)	118 (70.2)	168 (71.2)
Frequency of meal per day	≥4	26 (25.7)	75 (74.3)	101 (42.8)
	≤3 time	39 (28.9)	96 (71.1)	135 (57.2)
Taking meat at least once a month	Yes	20 (18)	91 (82)	111 (47)
	No	45 (36)	80 (64)	125 (53)
Taking egg at least once a month	Yes	27 (23.7)	87 (76.3)	114 (48.3)
	No	38 (31.1)	84 (68.9)	122 (51.7)
Taking milk at least once a month	Yes	34 (28.8)	84 (71.2)	118 (50)
	No	33 (28)	85 (72)	118 (50)
MUAC in cm	≥21	42 (21.9)	150 (78.1)	192 (81.35)
	<21	23 (52.3)	21 (47.7)	44 (18.65)
IP infection	No	45 (24.1)	142 (75.9)	187 (79.24)
	Yes	20 (40.8)	29 (59.2)	49 (20.76)

MUAC: mid-upper arm circumference, IP: intestinal parasite, and IPG: interpregnancy gap.

**Table 4:** Bivariate and multivariate analysis of anemia and related factors among study participant in a Kulito Health Center, SNNPR, Ethiopia, 2018 (*n* = 236).

Variable		Hemoglobin status		COR (95% CI)	AOR (95% CI)
		Anemic *n* (%)	Normal *n* (%)		
Age (years)				*P* ≤ 0.047	0.243
	16–24	25 (31.3%)	55 (68.8%)	1	1
	24–32	22 (20.2%)	87 (79.8%)	0.556 (0.286–1.082)	0.749 (0.192–2.921)
	32–42	18 (38.3%)	29 (61.7%)	1.366 (0.642–2.904)	1.730 (0.341–8.772)

Occupation				*P* ≤ 0.016	0.355
	Employed	19 (19.2%)	80 (80.8%)	1	1
	Unemployed	46 (33.6%)	91 (66.4%)	2.128 (1.153–3.929)	1.514 (0.628–3.646)

Educational status				*P* ≤ 0.089	0.186
	Primary	38 (24%)	120 (76%)	1	1
	Illiterate	27 (34.6%)	51 (65.4%)	1.672 (0.925–3.023)	0.504 (0.183–1.391)

Family income				*P* ≤ 0.062	0.586
	≥2000 birr	18 (20.7%)	69 (78.3%)	1	1
	<2000 birr	47 (31.5%)	102 (68.5%)	1.810(0.971–3.374)	1.349 (0.459–3.963)

Family size (people)				*P* ≤ 0.130	0.459
	≤4	34 (23.9)	108 (76.1)	1	1
	≥5	31 (33)	63 (67)	1.563(0.877–2.785)	0.638 (0.194–2.096)

Residency				*P* ≤ 0.002	0.137
	Urban	28 (20%)	112 (80%)	1	1
	Rural	37 (38.5%)	59 (61.5%)	2.508 (1.400–4.496)	1.878 (0.818–4.310)

Gravidity				*P* ≤ 0.013	0.053
	≤3	38 (23.45%)	124 (75.55)	1	1
	≥4	27 (36.5%)	47 (63.5)	1.931 (1.062–3.511)	2.197 (0.990–4.876)

Gestational age				*P* ≤ 0.044	0.060
	1^st^ trimester	4 (10.3%)	35 (89.7)	1	1
	2^nd^ trimester	21 (30%)	49 (70)	3.750 (1.183–11.889)	3.868 (0.696–21.485)
	3^rd^ trimester	40 (31.5%)	87 (68.5)	4.023 (1.339–12.087)	6.457 (1.276–32.729)^*∗*^

IPG (years)				*P* ≤ 0.018	0.013
	≥2	23 (22.1%)	81 (77.9%)	1	1
	<2	20 (40.8%)	29 (59.2%)	2.429 (1.166–5.061)	2.863 (1.245–6.582)^*∗*^

Taking iron folate				*P* ≤ 0.232	0.755
	Yes	15 (22.1%)	53 (77.9%)	1	1
	No	50 (29.8%)	118 (70.2%)	1.497 (0.772–2.902)	1.166 (0.444–3.066)

Taking meat at least once a month				*P* ≤ 0.003	0.322
	Yes	20 (18%)	91 (82%)	1	1
	No	45 (36%)	80 (64%)	2.500(1.363–4.584)	1.618 (0.625–4.194)

Taking egg at least once a month				*P* ≤ 0.174	0.742
	Yes	27 (23.7%)	87 (76.3%)	1	1
	No	38 (31.1%)	84 (68.9%)	1.492 (0.838–2.658)	0.857 (0.342–2.147)

MUAC in cm				*P* ≤ 0.0001	0.040
	≥21	42 (21.9%)	150 (78.1%)	1	1
	<21	23 (52.3%)	21 (47.7%)	3.912 (1.975–7.747)	2.595 (1.044–6.450)^*∗*^

IP infection				*P* ≤ 0.021	0.604
	No	45 (24.1%)	142 (75.9%)	1	1
	Yes	20 (40.8%)	29 (59.2%)	2.176 (1.124–4.235)	1.328 (0.454–3.885)

^*∗*^Statistically significant at 95% CI, *P* value < 0.05; 1-reference, CI: confidence interval, and AOR: adjusted odds ratio.

**Table 5:** The relationship between anemia and HP infection among study participants in Kulito Health Center, SNNPR, Ethiopia, 2018 (*n* = 236).

Variable		Hemoglobin status		COR (95% CI)	AOR (95% CI)
		Anemic *n* (%)	Normal *n* (%)		
HP infection				*P* ≤ 0.001	0.008
	No	18 (16.8%)	89 (83.2%)	1	1
	Yes	47 (36.4%)	82 (63.6%)	2.834 (1.524–5.271)	3.064 (1.336–7.027)^*∗*^

^∗^Statistically significant at 95% CI, P value < 0.05.

## Data Availability

The original data for this study are available from the corresponding author on reasonable request.
